# MRA-free intracranial vessel localization on MR vessel wall images

**DOI:** 10.1038/s41598-022-10256-2

**Published:** 2022-04-14

**Authors:** Weijia Fan, Yudi Sang, Hanyue Zhou, Jiayu Xiao, Zhaoyang Fan, Dan Ruan

**Affiliations:** 1 Department of Physics, University of California, Los Angeles, Los Angeles, CA USA; 2 Department of Bioengineering, University of California, Los Angeles, Los Angeles, CA USA; 3 Department of Radiology, University of Southern California, Los Angeles, CA USA; 4 Department of Radiation Oncology, University of Southern California, Los Angeles, CA USA; 5 Department of Biomedical Engineering, University of Southern California, Los Angeles, CA USA; 6 Department of Radiation Oncology, University of California, Los Angeles, Los Angeles, CA USA

**Keywords:** Medical imaging, Biomedical engineering

## Abstract

Analysis of vessel morphology is important in assessing intracranial atherosclerosis disease (ICAD). Recently, magnetic resonance (MR) vessel wall imaging (VWI) has been introduced to image ICAD and characterize morphology for atherosclerotic lesions. In order to automatically perform quantitative analysis on VWI data, MR angiography (MRA) acquired in the same imaging session is typically used to localize the vessel segments of interest. However, MRA may be unavailable caused by the lack or failure of the sequence in a VWI protocol. This study aims to investigate the feasibility to infer the vessel location directly from VWI. We propose to synergize an atlas-based method to preserve general vessel structure topology with a deep learning network in the motion field domain to correct the residual geometric error. Performance is quantified by examining the agreement between the extracted vessel structures from the pair-acquired and alignment-corrected angiogram, and the estimated output using a cross-validation scheme. Our proposed pipeline yields clinically feasible performance in localizing intracranial vessels, demonstrating the promise of performing vessel morphology analysis using VWI alone.

## Introduction

Intracranial atherosclerosis disease (ICAD) is a common cause of ischemic stroke in the United States and worldwide^1,2^. Luminal imaging approaches, such as computed tomographic angiography (CTA) and magnetic resonance angiography (MRA), are routinely used for the diagnosis of ICAD by detecting arterial lumen stenosis and evaluating stenotic severity. However, high-risk atherosclerotic lesions may present with a large plaque burden in the vessel wall in the absence of severe luminal stenosis^3^.

MR vessel wall imaging (VWI) is a non-invasive approach to directly image the vessel wall of large intracranial arteries. It has been shown to be useful in characterizing ICAD lesions by providing qualitative or quantitative morphological measurements^4–6^. There are recent efforts to automate VWI analysis for improved efficiency and consistency^7^. MRA that is acquired in the same imaging session is typically used to localize the vessel segments of interest. However, there is a chance of the MRA sequence missing due to scan failure or significant displacement between the VWI. Another disadvantage of using the time-of-flight (TOF) MRA is the prolonged acquisition time^8^. Therefore, it would be highly desirable if pre-contrast VWI could be used alone without MRA, to reduce complexity and time cost.

**Figure 1:**
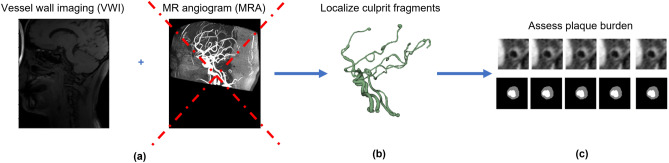
Schema for proposed modification and its impact on the current workflow for vessel wall imaging. Typical workflow for quantitative plaque assessment (**a**) acquires both VWI and MRA in the same session, (**b**) extracts and locates vessel segments of interest, and (**c**) identifies the corresponding region in VWI, performs detailed lumen, vessel wall segmentation to quantify plaque burden, and identifies the most stenotic slice. This study investigates the feasibility to rid the MRA module in this procedure.

This study addresses this challenging need for the first time and investigates the feasibility of localizing intracranial blood vessels using VWI directly. Figure 1 illustrates the envisioned modification to the current workflow.

## Method

### Initial investigation, identification of bottleneck, and overall design rationale

The most direct approach to label vessels is by segmentation. Typical segmentation methods include thresholding, clustering, edge-based detection^9,10^, or deep learning methods using fully convolutional network (FCN), U-Net, and SegNet, etc.^11–14^.

An alternative is to mimic clinical workflow by synthesizing an intermediate MRA-like image, hoping that this synthesis would alleviate the segmentation challenge. Synthesis has been used to either transfer information across subcategories within the same general modality, such as different dose levels for PET^15^ or CT^16,17^, different beam geometry in CT vs CBCT^18^, different field in MRI^19^, or across different imaging modalities amongst CT, MR, and PET^20–22^. Synthesis can be performed either with direct modeling^23^ or data-driven learning using Convolutional Neural Networks (CNN)^24^, with variations including U-Net^25,26^, or various adversarial logics^15,19,27,28^.

A less popular approach nowadays is the atlas-based approach, where the new test input is registered to a set of atlases with known labels, and the atlas labels are populated to the test coordinate, with possible fusion^29^, to create the test label estimate. Major factors affecting the efficacy of atlas-based approaches include atlas selection^30–32^, registration approach and setup^33^, and fusion scheme^33,34^. The central registration process is often performed in an optimization setting, where the deformation vector field (DVF) is sought to maximize an alignment metric. Multi-resolution or hierarchical schemes are commonly used to enhance the robustness to local optimality^35^. Recently, deep networks have been developed to perform registration in either supervised^36,37^ or unsupervised fashions^38–40^. Scale adaptation, auxiliary structure labels, and sophisticated priors have been adopted to further improve registration performance^41–44^*.*

Atlas-based approach has the benefit of being more interpretable and may be better controlled by imposing complexity conditions on the registration modules. Furthermore, adopting a general atlas-based paradigm does not necessarily exclude the use of certain deep learning techniques, as we will demonstrate with our design in this study.

**Figure 2:**
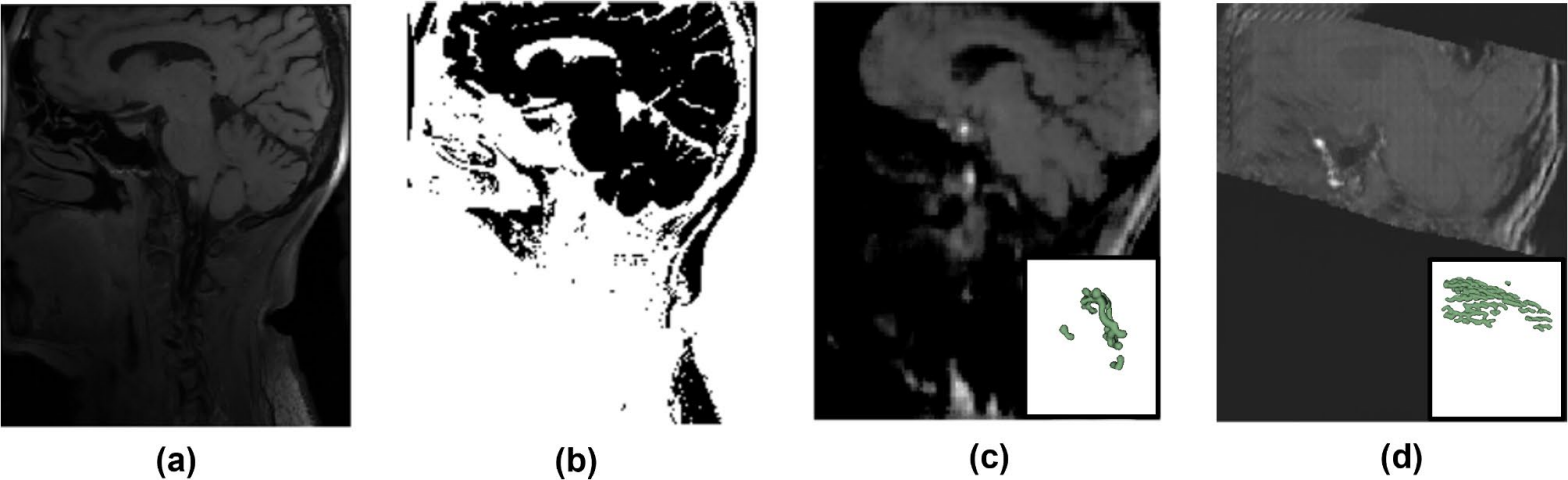
Challenges with vessel identification and failure of common direct segmentation or synthesis approaches. (**a**) VWI has low contrast and heterogenous signal strength, challenging vessel segmentation; (**b**) the Otsu algorithm fails to differentiate vessels from rest of the brain; the MRA-like image synthesized by (**c**) U-Net and (**d**) GAN all result in fragmented and inconsistent vessel segmentation.

Figure 2 shows examples of preliminary test results using typical Otsu’s thresholding, U-Net, and GAN for vessel synthesis and segmentation. It can be observed that the lack of consistent contrast between the black blood vessels and the surrounding tissues, the ambiguity in interpreting low intensity values, and the strong appearance heterogeneity and inconsistency across different scans had virtually rendered these results useless. Even if certain vessel segments were rendered correctly, it is hard to interpret them confidently without any larger-scale vessel structure context. While it is possible that a more advanced acquisition in combination with investigational post-processing may render VWI of higher quality and consistency in order to work with direct synthesis methods^26^, it is important to avail the large community with a working approach compatible with the typical, less-demanding, and clinically-accessible scanning protocol.

**Figure 3:**
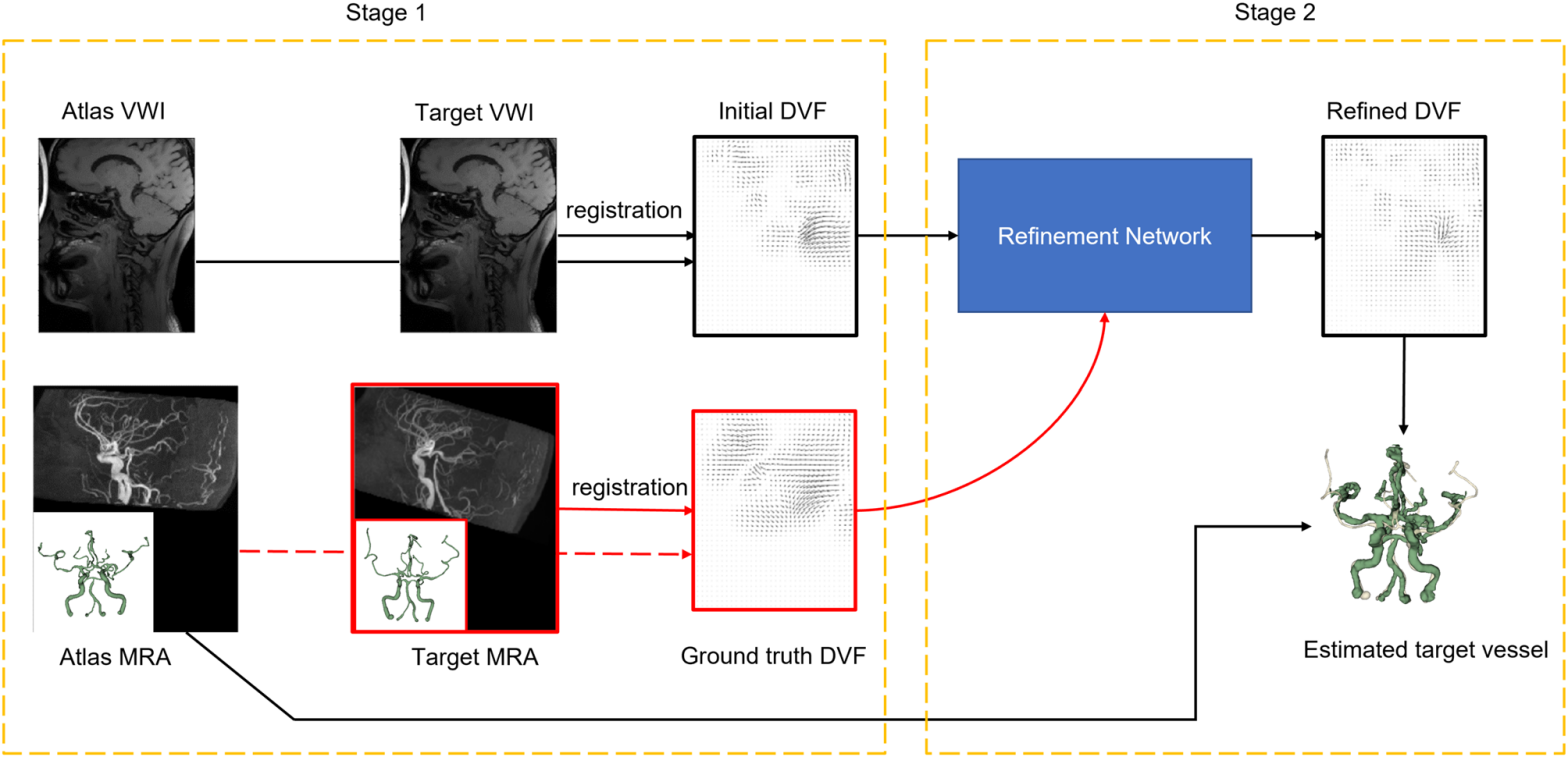
Design rationale. The two-stage pipeline consists of using conventional B-spline model for coarse registration and a deep-learning based refinement network for correcting registration error. Stage 1 is based on classic well-controlled registration, while stage 2 utilizes a deep network for registration refinement to map VWI-derived DVF to MRA-derived DVF. Red frames in the schema indicates modules only available and used during training.

Therefore, we propose to develop the vessel localization with a registration core in an atlas-based setting, so that the vasculature system integrity is implicitly “imposed” by that of the atlas, in combination with a controlled DVF estimate, as shown in Fig. 3. In particular, we propose a two-stage sequential pipeline: stage one performs a coarse registration with a low degree of freedom to both set up the general correspondence and identify the most relevant atlases; and stage two performs DVF refinement to correct for residual registration error. Stage one uses a model-driven approach for complexity control and regularization, and stage two uses a customized deep learning network where the magnitude of the adjustment is controlled. We purposely use two different approaches (model-driven vs data-driven) in these two stages, so that errors may be considered as approximately uncorrelated, allowing us to train the stage two network and perform the corresponding inference without concerns for confounding behaviors from stage one. Finally, the post-correction DVF is applied to the vessel tree extracted from atlas MRA to localize the vessel structure for the target test case.

### Stage one: model-based low-complexity registration and adaptive atlas selection

For the test subject with VWI, affine registration is performed between the test VWI and the VWI of all atlas cases, and the post-rigid adjustment similarity is assessed. For each given target subject*,* a subset of the top matching atlases with high mutual information to the target VWI*,* and low degree-of-freedom B-spline-based deformable registration is performed between each atlas in this subset and the target test VWI. In this way, the atlases are customized to each subject: if the input is a normal subject, it is highly possible that the selected atlases are also normal; on the other hand, if the subject exhibits occlusion, then chances are that the relevant atlases have occlusion presence. The initial rigid transformation and the subsequent B-spline parameterized deformation are combined to generate a DVF for each of the selected atlas and take its aligned VWI and MRA simultaneously to the test subject coordinate.

### Stage two: deep learning-based refinement for DVF residual correction

A supervised deep learning neural network is designed for correcting the residuals in DVF from the low complexity deformable registration in stage one. During training, the target MRA is known, and we generate the “ground truth” DVF by performing an intensity-based deformable registration between the atlas MRA and the target MRA. While we realize the limited quality of this vector field estimate, vessel boundaries coincide with high local MRA contrast, where high accuracy is expected from the deformable registration. Therefore, such DVF suffices for the purpose of warping the atlas vessels to estimate the target one. In particular, the high blood contrast in angiograms automatically focuses non-rigid registration matching effort to vessel neighborhoods. We develop and investigate two different correction network models—the DVF2∆DVF and IM2∆DVF model—they share a common network structure with different specific configurations.

#### Common network scheme

**Figure 4:**
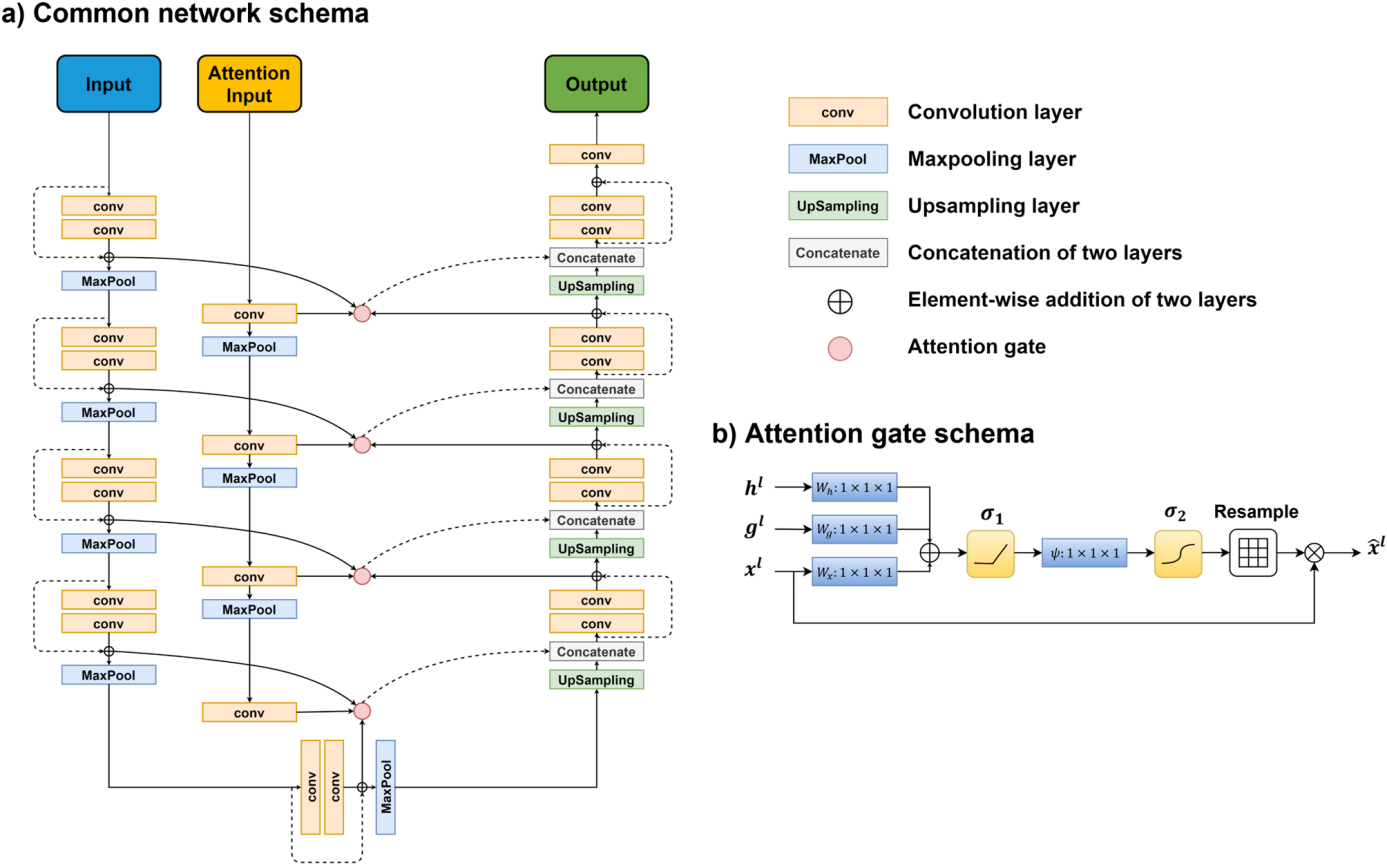
Common network scheme. (**a**) Common network structure for estimating the residual DVF using U-Net backbone with residual blocks and an auxiliary attention path. (**b**) The schema for attention gate at each block, with h representing gate signal from encoding pathway, g representing gate signal from auxiliary attention path, and x representing the layer at decoding pathway. The resulted $\hat{x}$ will be concatenated to the decoding pathway.

Both DVF2∆DVF and IM2∆DVF models use a common network structure consisting of a 3D U-Net backbone^25,45^ with residual blocks^46,47^ and an attention pathway^48^. The soft attention mechanism is employed to encourage the network to focus on local regions with greater errors. We modify the attention gate in the U-Net so that in addition to the inherited information from the corresponding layer in the decoding pathway, an auxiliary attention path is introduced, as shown in Fig. 4. The input from the auxiliary attention pathway is gradually down-sampled, alongside the encoding pathway in the U-Net backbone. In the modified ResUnet, in each encoding unit, two 3D convolution layers of kernel size 3 are followed by one maxpooling layer to down-sample the volume by a scale of 2. The number of filters starts at 16 in the first layer and doubles in each subsequent encoding unit. In each decoding unit, an upconvolution layer of kernel size 2 is followed by two convolution layers of kernel size 3. The skip connections are used both within blocks and between encoding–decoding pathways. Within each block, the input before the first convolution layer is added voxel-wise to the output of the second convolution layer. The output of each encoding unit, after passing through the attention gate with the attention information, is concatenated to the corresponding unit in decoding pathway. One 1 × 1 × 1 convolution layer is used at last to recover the three-channel result. ReLU activation is used in each convolution layer except the last one.

Parallelogram masks from the stage one affine transformation indicating the region of interest (ROI) in angiograms are provided to the network from additional input channels. The mean-squared discrepancy between the predicted DVF residual and ground truth residual in the masked region is used as the training objective.

#### Two different DVF refinement structures

**Figure 5:**
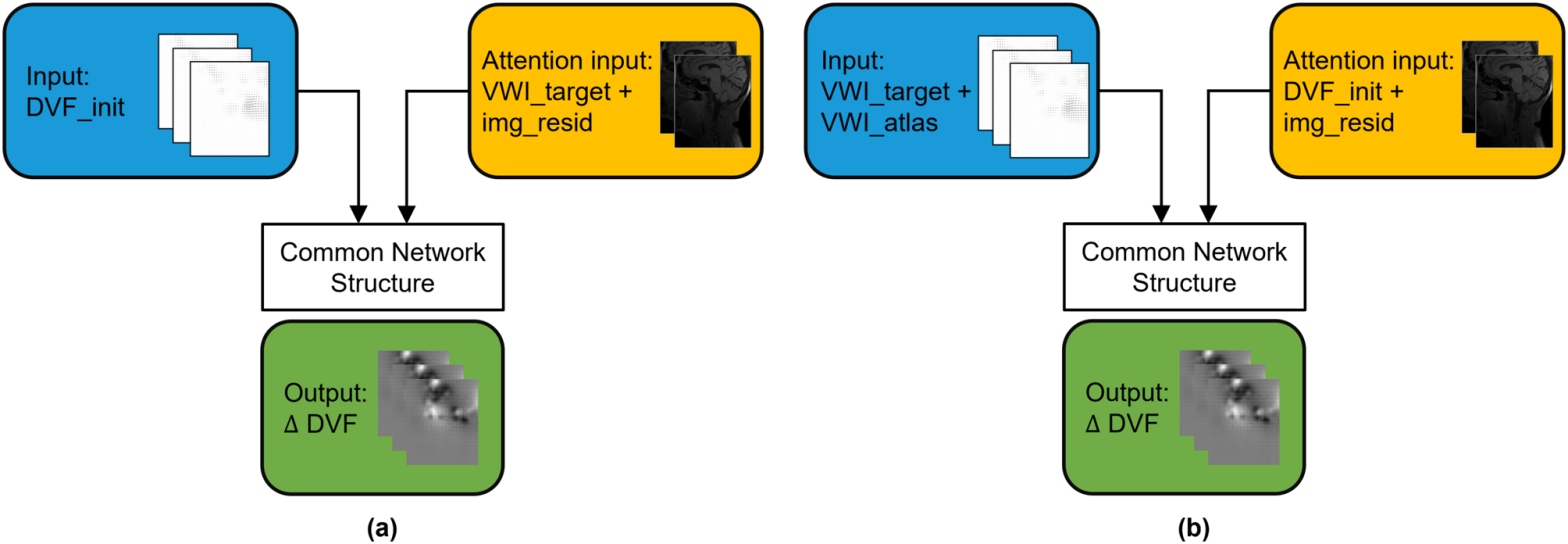
Two DVF refinement structures. (**a**) DVF2∆DVF with DVF input and images to drive attention; (**b**) Im2∆DVF with image input and DVF as well as residual error from stage 1 to drive attention.

Figure 5 illustrates the two different DVF refinement structures both utilizing the common attention-enhanced network structure shown in Fig. 4. Figure 5a shows the DVF2∆DVF pipeline where the initial DVF from deformable registration is taken as the U-Net input, and the residual between original DVF and ground truth DVF is the output. The target VWI, and intensity difference between the target VWI and the atlas VWI warped by initial DVF are used as the input to the attention pathway. The IM2∆DVF network in Fig. 5b uses the stacked target and atlas VWI as the input, and the residual between original DVF and ground truth DVF as the output. The original DVF, along with the intensity difference between the target VWI and the atlas VWI warped by initial DVF, is input into the attention pathway.

### Hybrid atlas integration for individual segments

Using a single deformed atlas is susceptible to estimation artifact from inter-subject anatomical variation and registration uncertainty, and it is desirable to integrate the inferred vessel structure from multiple atlases. However, it is nontrivial to perform fusion on a whole vessel tree without sacrificing anatomical geometry. In clinical tasks, it is typical that a local fragment is of interest, and it could be beneficial to choose an atlas based on the ROI. For each segment candidate specified by the clinician, local normalized cross-correlation is evaluated between the target VWI and each warped atlas, and the one corresponding to the highest correlation is used to render the local fragment estimate.

## Data description and experiment details

### Dataset

Under Cedar-Sinai Institutional Review Board approval #Pro00055925, a retrospective deidentified dataset containing VWI and MRA images from 30 subjects was used in this study. The need for informed consent was waived, and all methods were carried out in accordance with relevant guidelines and regulations. The images had been acquired on a 3.0 T MRI scanner (MAGNETOM Prisma, Siemens Healthineers, Germany) with a 64-channel head-neck coil. The imaging protocol consisted of 3D time-of-flight (TOF) MRA and pre-contrast MR-VWI. Imaging parameters for MR-VWI were as follows: repetition time, 900 ms; echo time, 15 ms; field of view, 170 × 210 mm2; 240 slices; voxel size, isotropic 0.55 mm; scan time, 8 min. An experienced clinician identified segments for possible plaque presentations in the basilar artery (BA), left or right vertebral artery (LVA/RVA), left or right middle cerebral artery (LMCA/RMCA), and left or right internal carotid artery (LICA/RICA).

### Experimental details

All registration other than the refinement was performed using the SimpleITK toolkit^49^. 3D rigid registration was first performed between paired (MRA, VWI) volumes to transform MRA to the VWI coordinate. Offset parameters were initialized to zero and mutual information was maximized with a multi-resolution scheme. Three-layer B-spline models with control points every 12 pixels were applied to perform the nonrigid registration.

For deep network based refinement, the image volume for each patient was acquired with 240 × 384 × 318 voxels. In order to save computational memory, the outer regions of the images without major vessels were excluded from the training dataset and the volumes were down-sampled. Each image volume was first cropped to 192 × 384 × 320 voxels and then rescaled to 48 × 96 × 80 voxels.

Vessel extraction was performed using a tubular filter based on local Hessian^50^. The input MRA images were first resampled to a higher resolution and manual seed points were provided at the interior of a vessel. The minimum roundness of the tube’s cross-section was set to 0.2 to reflect reasonable circular symmetry in most vessels. The scale for tube radius was estimated recursively by starting from a large candidate value of 1.5 mm until a tubular vessel was found. The vessel was incrementally grown, whereupon extraction of a tubular segment, its endpoints were added to the sets of seed points for the next iteration. A few rounds of scanning were repeated to capture finer tertiary branching.

Six-fold cross-validation has been performed on the acquired datasets. With 30 subjects in total and three atlases for each subject, 25 subjects were used for training and 5 subjects were used for testing each time. With the use of a Unet infrastructure to be context-conscious and given the spatially varying anatomy of intracranial vessel tree topology, we took a conservative option and did not perform any transformation-based augmentation. A GAN-type augmentation has the potential to be useful and we will investigate its role in future work*.*

### Performance evaluation

The accordance of vessels localized from VWI to the vessels extracted from corresponding ground-truth angiograms was assessed using one-direction Hausdorff Distance (HD). Given two point sets $GT = \left\{ {x_{1} , x_{2} , x_{3} , \ldots ,x_{n} } \right\}$ and $PRED = \left\{ {y_{1} , y_{2} , \ldots , y_{n} } \right\}$, representing the point sets for ground truth vessel structures and estimated vessel structures respectively, the one-direction Hausdorff Distance $H\left( {GT,PRED} \right)$ is defined as:

$$H\left( {GT,PRED} \right) = \mathop {\max }\limits_{x \in GT} \left( {\mathop {\min }\limits_{y \in PRED} \left| {\left| {y - x} \right|} \right|} \right),$$

and percentile HD is the percentile of the distribution for $\mathop {\min }\limits_{y \in PRED} \left| {\left| {y - x} \right|} \right|.$ For our evaluation, 80% HD, 90% HD, and 100% HD were assessed on each case. For each subject, four segments that may potentially present plaque or abnormal morphology were identified by an experienced clinician. Percentile HD metrics were also assessed on each of the four segments for local evaluation.

A clinical validation test was also performed on the estimated vessels. In the clinical pipeline in Fig. 1, the estimated vessels are intended to track the vessel centerline to render cross-sectional views that will be used as input for segmentation. Therefore, the deviation between the centerline of estimated vessel tubes and that of the ground truth vessel tubes should be small enough for automatic cross-section generation and a maximal tolerance of 10 mm is a reasonable threshold. We assessed the odds of successfully accomplishing this task for each segment.

## Results

Root-mean-squared-error (RMSE) and mean-absolute-error (MAE) are defined in the following equations for each image volume of dimension $n_{1} \times n_{2} \times n_{3}$, and were used to evaluate the result of six-fold cross-validation, in the unit mm.

$$RMSE = \sqrt {\frac{{\mathop \sum \nolimits_{i} \left( {\left( {dx_{1}^{i} - dx_{2}^{i} } \right)^{2} + \left( {dy_{1}^{i} - dy_{2}^{i} } \right)^{2} + \left( {dz_{1}^{i} - dz_{2}^{i} } \right)^{2} } \right)}}{{n_{1} \times n_{2} \times n_{3} }}}$$

$$MAE = \frac{{\mathop \sum \nolimits_{i} \sqrt {\left( {dx_{1}^{i} - dx_{2}^{i} } \right)^{2} + \left( {dy_{1}^{i} - dy_{2}^{i} } \right)^{2} + \left( {dz_{1}^{i} - dz_{2}^{i} } \right)^{2} } }}{{n_{1} \times n_{2} \times n_{3} }} ,$$

where $dx_{1}^{i}$, $dy_{1}^{i}$, $dz_{1}^{i}$ are the predicted ∆DVF residual in $x, y, z$ direction for voxel $i$, while $dx_{2}^{i}$, $dy_{2}^{i}$, $dz_{2}^{i}$ are the corresponding ground truth ∆DVF.

Cross-validation shows that registration performance in RMSE and MAE (both in mm) are 6.15 ± 3.35 and 3.86 ± 2.05 before any refinement, and they reduce to 5.70 ± 2.57 and 4.37 ± 1.68 using the DVF2∆DVF refinement network, and to 5.05 ± 2.58 and 3.62 ± 1.63 using the IM2∆DVF refinement network. Two-sample t-tests have been performed of the results before any refinement network both with that after DVF2∆DVF refinement network and with that after IM2∆DVF refinement network. The resulted p-values are 0.31 and 0.01 on RMSEs, and are 0.07 and 0.39 on MAEs.

Table 1 shows the distribution of 80% HD, 90% HD, and 100% HD over the whole vessel structure for all deformed atlas vessels after registration only, after Im2∆DVF network, and after DVF2∆DVF network. The difference between the performance of these two models is very minimal.

**Table 1:** Distribution of 80% HD, 90% HD, and 100% HD evaluated between the whole estimated vessel structure and ground truth vessel structure. (unit: mm).

Metric	80% HD			90% HD			100% HD		
Model	Reg Only	DVF2∆DVF	Im2∆DVF	Reg Only	DVF2∆DVF	Im2∆DVF	Reg Only	DVF2∆DVF	Im2∆DVF
mean	6.15	6.16	6.34	11.90	11.20	11.48	33.53	32.82	30.00
std	3.58	3.03	2.84	5.95	5.41	5.43	9.02	8.39	8.38
min	1.73	2.54	3.19	3.54	4.67	4.41	13.90	18.80	19.94
25%	3.84	4.42	4.99	7.43	7.36	7.62	27.36	25.82	27.32
50%	4.95	5.36	5.55	10.33	11.04	10.94	33.59	31.53	32.37
75%	7.36	6.56	7.15	14.63	13.13	13.80	40.02	39.09	38.13
max	18.44	17.17	18.41	29.58	28.21	29.85	60.83	51.00	51.11

**Figure 6:**
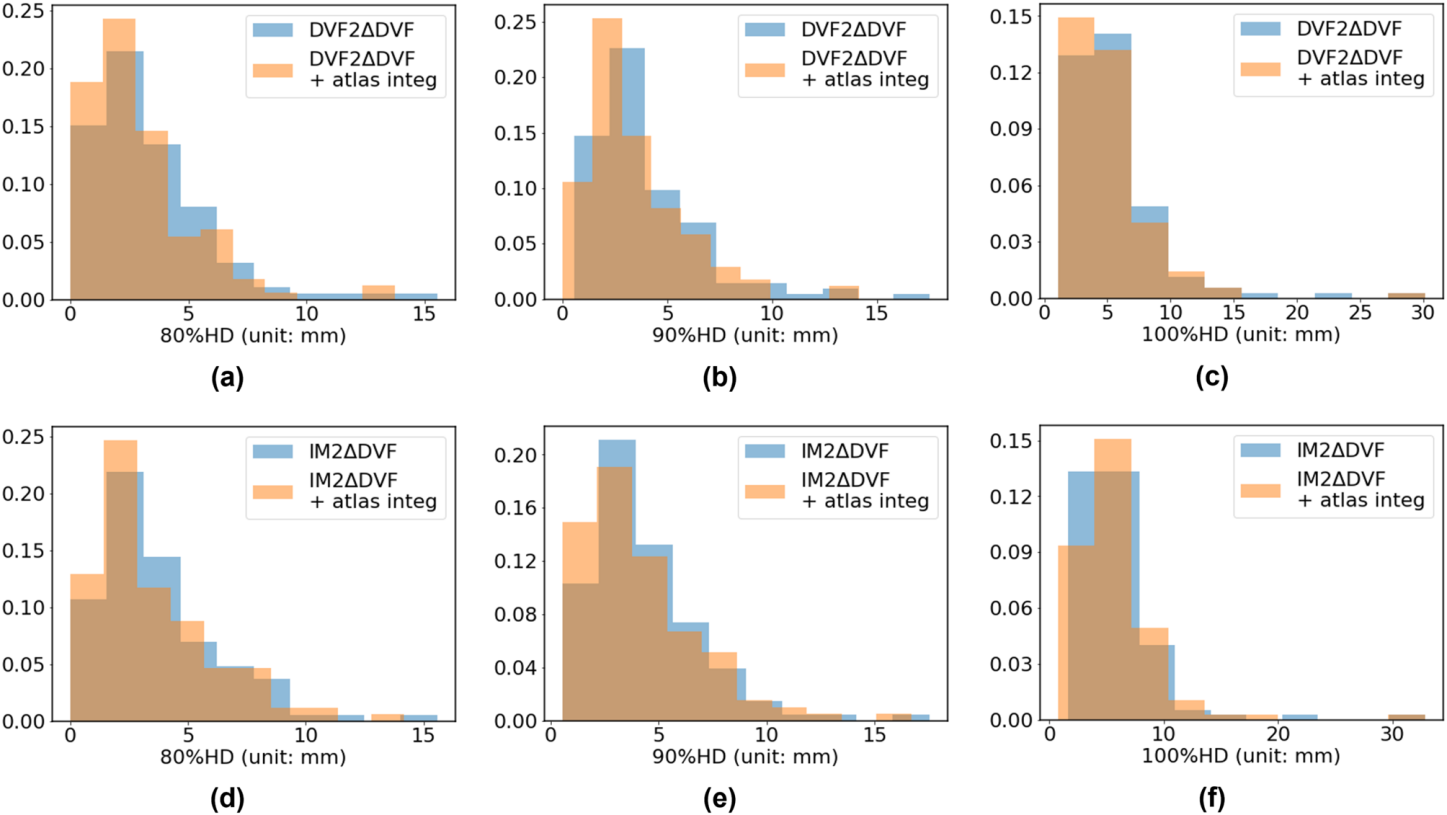
Vessel estimation accuracy when using a segmentation. The distribution of 80% HD, 90%HD, and 100% HD between the estimated vessel structure and the ground truth vessel structure before and after the DVF2∆DVF (**a**-**c**) or IM2∆DVF (**d**-**f**) refinement network. The distribution of 80% HD values for using no refinement network, using DVF2∆DVF, using DVF2∆DVF + atlas integration, using IM2∆DVF, and using IM2∆DVF + atlas integration are 2.92 ± 2.68, 3.33 ± 2.60, 3.67 ± 2.55, 289 ± 2.30, 3.50 ± 2.54, respectively. The distribution of 90% HD evaluated for these methods are 3.51 ± 2.87, 3.94 ± 2.76, 4.30 ± 2.69, 3.48 ± 2.40, and 4.14 ± 2.75, respectively. And the distribution of 100% HD evaluated for these methods are 5.12 ± 3.82, 5.54 ± 3.73, 5.98 ± 3.80, 5.10 ± 3.38, 5.83 ± 3.84, respectively.

Figure 6 shows the distribution of 80% HD, 90% HD, and 100% HD for the results from the top atlas and the results from atlas integration. The blue bars in each graph are the distribution of the metric after a refinement network (IM2∆DVF or DVF2∆DVF), while orange bars indicate the distribution of the same metric after that refinement network + atlas integration. Comparing the blue and orange bars in each subplot, we observe that with atlas integration, the distribution of the metric has a smaller mean value, indicating that atlas integration would result in better accuracy of estimating vessel segments.

The percentages of valid segments upon clinical tests after no refinement network, using DVF2∆DVF for correction, using DVF2∆DVF + atlas integration method, using IM2∆DVF for correction, and using IM2∆DVF + atlas integration method are 75.8 ± 7.4%, 79.2 ± 5.8%, 86.7 ± 7.5%, 77.5 ± 8.8%, and 84.2 ± 9.7%, respectively. Two-sample t-tests have been performed between the result from no refinement network and the result after each other method, and the resulted p-values are 0.41, 0.03, 0.73, and 0.13.

**Figure 7:**
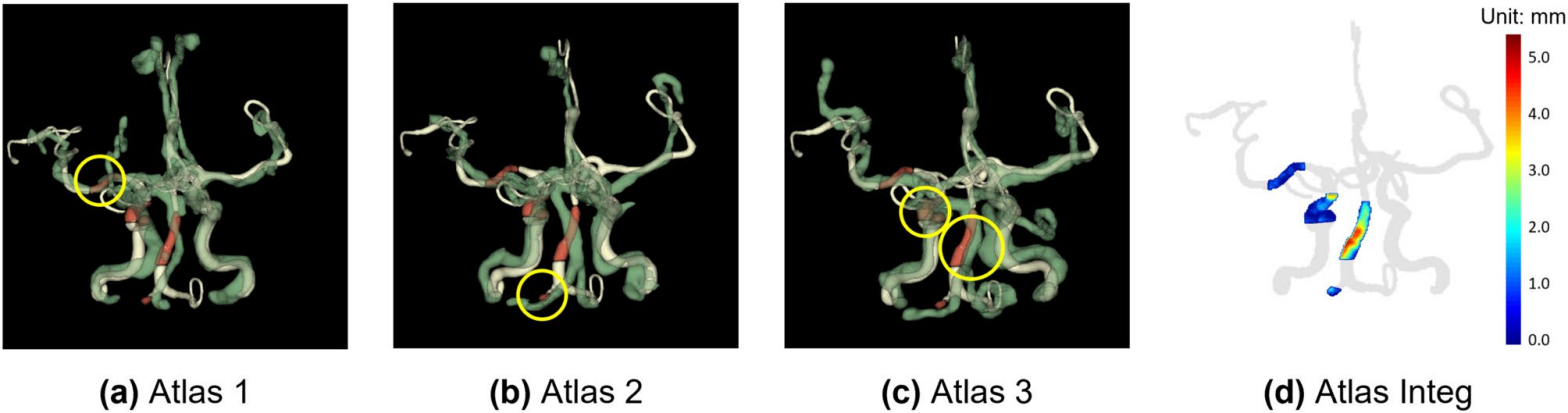
An example case illustrating atlas integration. The target vessel structure (white), with four clinically significant vessel segments (red), is overlapped with top three estimated vessel structures from atlas (green). The yellow circles highlight the chosen segment estimate(s) from each atlas. In this specific example, (**a**) the right middle cerebral artery (RMCA) was obtained from atlas 1, (**b**) the right vertebral artery (RVA) was obtained from atlas 2, and (**c**) the right internal carotid artery (RICA) and basilar artery (BA) were obtained from atlas 3. (**d**) The resulted vessel information has smaller than 5 mm deviation from the ground truth vessel on all segments.

Figure 7 shows an illustrating example case where taking segments from different atlas sources clearly improve local segment identification accuracy. The estimated vessel structure has universally less than 5 mm deviation from the ground truth on all segments, successfully differentiating the target vessel fragment from any adjacent vessel structure, supporting subsequent proper rendering of cross-sectional views for further quantitative analysis of plaque characteristics.

## Discussions and conclusions

Registration errors in both RMSE and MAE have been reduced by both refinement networks. In particular, IM2∆DVF refinement network has reduced RMSE by 1 mm, with statistical significance. Examining 80%, 90% and 100% HD metrics alone reveal only moderate improvement by introducing the refinement network. However, when considering the performance in the clinical context of valid vessel localization and detection, DVF2∆DVF network with a simple atlas integration has increased the success rate from 76 to 87%, with statistical significance. This shows that while RMSE and MAE for ∆DVF, and Hausdorff distances for set accordance are reasonable surrogates, our proposed methods may behave differently from the intended clinical endpoint.

While it is possible that a more advanced acquisition in combination with investigational post-processing may render VWI of higher quality and consistency to work with direct synthesis methods^26^ that overcomes the observed limitations, the method developed in this work is compatible with common, less-demanding, and clinically-accessible scanning protocol.

Our results, though still have room for improvements, have shown a first breakthrough to generate morphologically robust and clinically usable vessel identification. It provides a coarse-level localization to generate attention to the segment of interest and feeds it into the subsequent plaque quantification analysis. It serves the purpose of rough identification and exclusion of disruptive textures such as excessive bifurcations or adjacent vessels. The achieved sub-cm accuracy suffices those purposes. Typical subsequent vessel segmentation development usually involves augmentation methods that mitigate strict requirements on precise centerline identification or tracing^51–53^.

In short, the proposed combination of atlas-based framework manages to regulate vessel topology while preserving the target’s specific appearance, and the deep correction network on the DVF domain further accounts for DVFs heterogeneity and focal vessel performance for each target case. Although there are remaining geometric residuals in precise labeling of intracranial vessel voxels, the proposed approach achieves promising results for vessel localization to support cross-sectional rendering for further plaque quantification. We are actively working on integrating the current module into an analytical pipeline to perform end-to-end analysis, and conducting ablation studies and statistical analysis in a multi-institutional validation setting^51,54^.

## Data Availability

The datasets generated during and/or analyzed during the current study are available from the corresponding author on reasonable request.
